# Surface cues shape procoagulant properties of amyloidogenic microclots

**DOI:** 10.1038/s41419-026-08884-x

**Published:** 2026-06-02

**Authors:** Etheresia Pretorius, Chantelle Venter, Massimo Nunes, Alain R. Thierry, Douglas B. Kell

**Affiliations:** 1 Department of Physiological Sciences, Faculty of Science, Stellenbosch University, Stellenbosch, South Africa; 2 Department of Biochemistry, Cell and Systems Biology, Institute of Systems, Molecular and Integrative Biology, Faculty of Health and Life Sciences, University of Liverpool, Liverpool, UK; 3 IRCM, Montpellier Cancer Research Institute, INSERM U1194, Montpellier University, Montpellier, France; 4 ICM, Institut Régional du Cancer de Montpellier, Montpellier, France

**Keywords:** Thrombosis, Diagnostic markers

## Abstract

Hypercoagulability, immunothrombosis, and protein misfolding are deeply interconnected processes that converge on cell membranes as central orchestrators of thrombo-inflammation. In health, membrane lipid asymmetry, intact glycocalyx, and regulated receptor activity maintain vascular homeostasis. During inflammation or cell death, however, phosphatidylserine (PS) externalization, protein unfolding, and damage to glycosaminoglycans expose negatively charged, amyloidogenic surfaces that attract coagulation factors, inflammatory mediators, and adhesion proteins. These events generate catalytic sites for prothrombinase assembly. We review how cellular debris, microparticles, immune complexes such as neutrophil extracellular traps, and amyloidogenic plasma proteins, including serum amyloid A, interact with fibrinogen to form circulating (heterogeneous) procoagulant complexes, we term fibrinaloid microclot complexes (FMCs). Distinct from canonical fibrin clots, these FMCs display β-sheet–rich features, ThT-binding, and resistance to fibrinolysis, implicating them as key drivers of vascular pathology in inflammatory (and post-viral) syndromes. Recognizing different FMC phenotypes, mechanisms, and biochemical composition of these circulating complexes provides new insights into the pathogenesis of systemic inflammatory diseases and clarifies how membrane damage, protein misfolding, and coagulation converge to drive thrombo-inflammation. These insights position FMCs as a conceptual framework for understanding pathological clotting across inflammatory and post-infectious vascular syndromes.

## Highlights

- Membrane damage as a procoagulant trigger: Loss of membrane integrity during apoptosis, necrosis, or senescence leads to phosphatidylserine externalization, glycosaminoglycan degradation, and protein unfolding. These changes expose negatively charged, amyloidogenic surfaces that act as catalytic hubs for coagulation and inflammation.
- Cell death–derived debris as catalytic scaffolds: Fragments and microparticles released from dying cells retain exposed PS and damaged proteins, recruiting fibrinogen, prothrombinase, and inflammatory molecules. This transforms normal cellular debris into persistent procoagulant seeding sites.
- Formation of fibrinaloid microclot complexes (FMCs): Circulating fibrin(ogen), serum amyloid A (SAA), von Willebrand factor (vWF), and other amyloidogenic proteins co-aggregate on damaged membranes, forming β-sheet–rich, Thioflavin T-positive, fibrinolysis-resistant fibrinaloid microclot complexes.
- Distinct from canonical thrombin clots: FMCs arise from protein-membrane misfolding interactions rather than thrombin activation. These amyloidogenic surfaces form heterogeneous catalytic platforms that amplify coagulation independently of traditional pathways.
- Cell death as the nexus of thrombo-inflammation: The continuum of membrane injury, from reversible stress to irreversible cell death and links inflammation, amyloid formation, and hypercoagulability. FMCs represent a structural fingerprint of this process and potential biomarkers and therapeutic targets.

## Introduction

It has long been recognized that hypercoagulability, immunothrombosis, and protein damage represent interconnected and complex pathophysiological processes [1–4]. The classical triggers of cellular injury and prothrombotic surface formation, together with the well-characterized coagulation cascades, culminating in the conversion of prothrombin to thrombin and fibrinogen to fibrin, are well understood [5]. Widespread vascular injury, characterized by endothelial disruption, platelet dysfunction, abnormalities of erythrocytes and white blood cells, and immune dysregulation at the cellular level, collectively drive hypercoagulability and immunothrombosis in conditions such as type 2 mellitus (T2DM), stroke, cardiovascular disease, and post-viral syndromes. In such conditions, endothelial cells and blood cells may undergo apoptosis or necrosis as a consequence of inflammatory cascades. At the center of these cell death processes is the cell membrane, which plays a fundamental role in orchestrating mechanisms of cell death [6, 7].

**Fig. 1:**
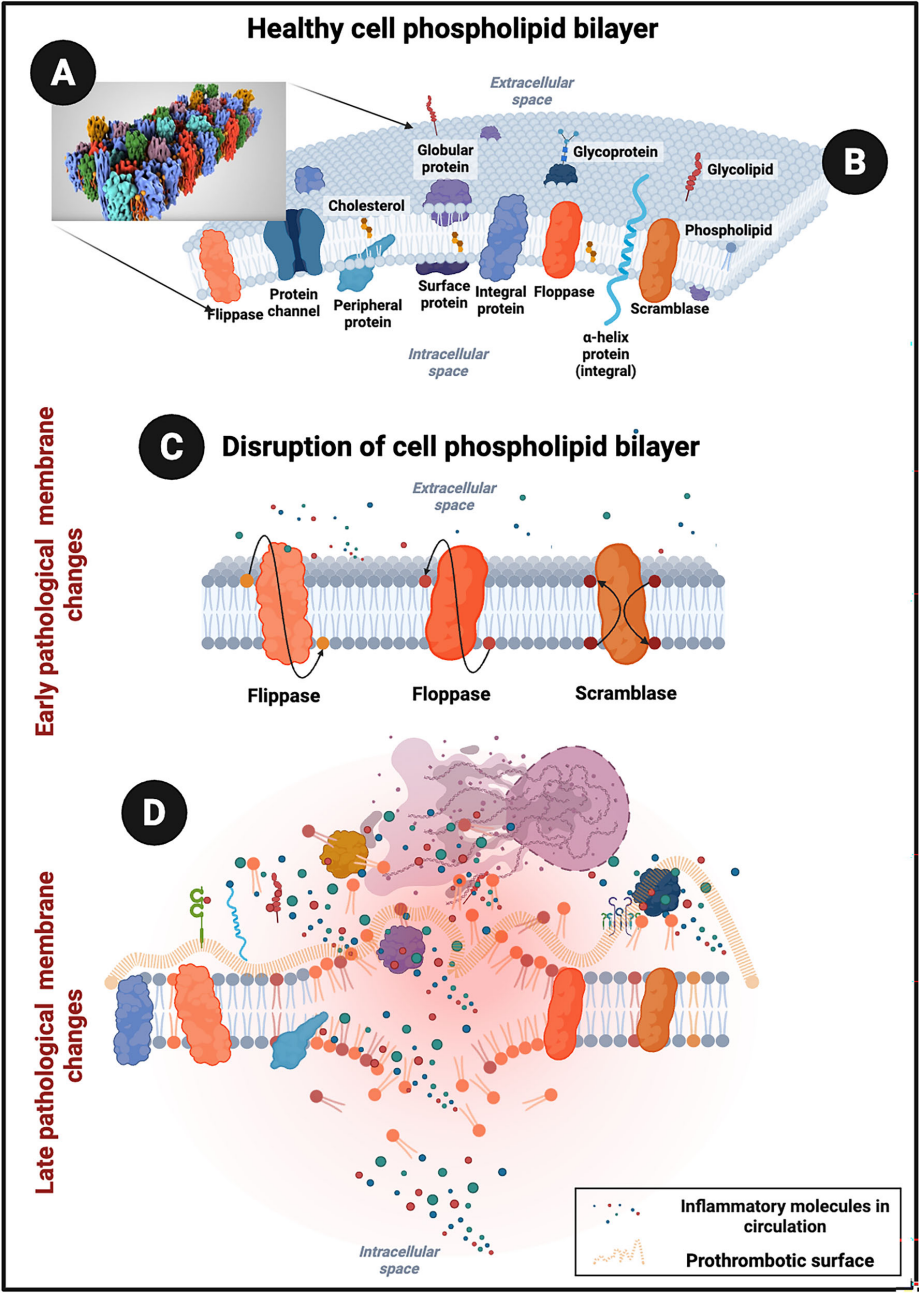
Membranes in health and disease. **A** Biochemical composition of membranes [11] (∼50:50 lipids versus proteins by area [12]). **B** The structure of a healthy cell membrane. In a physiologically intact cell, the lipid bilayer maintains asymmetry between inner and outer leaflets, and integral proteins are properly folded and distributed. This arrangement preserves selective permeability, electrochemical gradients, and homeostatic signaling. (Protein/lipid distribution not to scale). **C** Early membrane damage to form a prothrombotic surface. Oxidative stress, inflammatory mediators, or mechanical injury disrupt lipid asymmetry, expose phosphatidylserine and other negatively charged lipids, and induce clustering or unfolding of membrane proteins. These changes provide catalytic sites for coagulation reactions, contributing to early thrombo-inflammatory signaling. **D** Late stage membrane changes. Progressive damage leads to loss of membrane integrity, protein unfolding, and the release of microvesicles and cellular debris. These fragments expose negatively charged and amyloidogenic surfaces that bind inflammatory molecules, thereby serving as circulating procoagulant platforms and amplifying thrombo-inflammatory cascades. Created in https://BioRender.com.

The cell membranes of healthy endothelial cells, platelets, and blood cells consist of a phospholipid bilayer embedded with proteins that together ensure vascular integrity, immune surveillance, and hemostatic balance [8–10]. By weight, these membranes are close to a 50:50 ratio of proteins: lipids or more (and in many cases 3:1) [11, 12], although when counted by molecules, for every single protein molecule there may be as many as 50–100 molecules of lipid [13]. Despite being fewer in number, proteins are bulky and can occupy nearly half of the membrane surface area [12, 14], providing dynamic functions such as adhesion, signaling, and enzymatic activity [14, 15] (see Fig. 1).

Under normal conditions, phosphatidylserine (PS) is restricted to the inner leaflet of the bilayer by energy-dependent flippases, maintaining lipid asymmetry [16]. The ATP-dependent enzyme, flippase normally keeps PS inside the cell, but when a PS flop occurs in the opposite direction (Fig. 1C), PS acts as an ‘eat me’ signal on dead cells, and creates a scaffold for blood-clotting factors on activated platelets [17].

When endothelial cells are injured, when platelets become hyperactivated, or when red and white blood cells undergo stress, apoptosis, or eryptosis, scramblases are activated and PS also flips to the outer leaflet [17–21]. This externalization exposes a strongly negatively charged surface [22]. Although the total protein-to-lipid mass ratio remains unchanged, the exposure of negatively charged PS on the cell surface alters the biochemical landscape of the membrane [23–25]. Simultaneously with this PS flip, proteins are damaged. Protein damage and loss of proteostasis during cell stress exposes β-sheet motifs that can aggregate into amyloid-like assemblies [26]. This does not mean every dying cell becomes “amyloid-rich,” but it does mean that the risk of β-sheet aggregation increases sharply as proteins denature and unfold.

The flip of PS to the outer membrane leaflet has profound consequences for the protein–lipid balance of the cell surface. Because PS carries a negative charge, its externalization exposes new electrostatic sites that strongly influence how proteins bind, attracting integrins, annexins, and coagulation factors such as prothrombin and factor Xa, which effectively increases the functional protein content at the surface [27, 28]. In addition, PS redistribution alters lipid microdomains, promoting clustering of membrane proteins [29], including receptors and adhesion molecules [30], so that proteins occupy a larger proportion of the surface area even though their overall mass fraction within the membrane remains unchanged. This could create a shift in protein coverage, and in biophysical measurements it may appear that membranes are more protein-dense after PS exposure because cytoplasmic or plasma proteins are recruited to the newly exposed PS [31, 32]. In some instances, however, PS redistribution (externalization) on apoptotic or activated cells serves as an “eat-me” or tolerogenic signal [33] and also facilitates binding of serum proteins, opsonins, and complement. Lipid microdomains may shift with PS exposure, favoring receptor clustering and increasing functional protein coverage at the membrane, although direct evidence for that remains less well established.Specific examples illustrating this transformation, are known in platelets [34], where PS externalization drives assembly of the tenase and prothrombinase complexes [7, 35, 36], producing a strongly procoagulant phenotype [37]. A procoagulant environment is also seen in erythrocytes undergoing eryptosis [21]. PS exposure also serves as a clearance signal for macrophages [38]; while simultaneously attracting annexins, immunoglobulins, and complement proteins and in endothelial cells [39]. PS externalization during apoptosis or injury thus facilitates binding of clotting proteins, including prothrombin and factor Xa, thereby further shifting the membrane toward a protein-dominated interface [7, 28, 40]. Similarly, tissue factor (TF) is important for a procoagulant surface, as it is the principal initiator of one arm of the coagulation cascade. Under normal conditions, TF is largely confined to subendothelial tissues, where its separation from circulating blood maintains tight control of clotting activation. When vascular damage occurs, TF becomes exposed and rapidly engages circulating factor VII, triggering downstream steps of the coagulation cascade [41]. In pathological contexts, TF expression can also be upregulated in monocytes and macrophages in response to inflammatory mediators, including lipopolysaccharide, tumor necrosis factor-α, and interleukin-1. As a glycosylated membrane protein, TF requires interaction with PS for its full procoagulant activity [42, 43].

### Glycosaminoglyans

Glycosaminoglycans (GAGs), and in particular heparan sulfate proteoglycans (HSPGs) of the endothelial glycocalyx, are central regulators of both inflammation and thrombosis [44]. In the context of inflammation, GAGs modulate leukocyte adhesion, chemokine binding, and vascular barrier function [44–46]. They act as reservoirs for cytokines and chemokines, regulating their bioavailability and signaling. In thrombo-inflammation, intact GAGs help preserve anticoagulant activity by binding and activating proteins such as antithrombin and heparin cofactor II, while their degradation or shedding exposes prothrombotic endothelial surfaces, promotes leukocyte adhesion, and facilitates complement and coagulation activation [47, 48]. Loss of endothelial GAGs has been shown to directly trigger a procoagulant phenotype, whereas their preservation maintains vascular homeostasis [49]

### Creating a prothrombotic surface

When PS externalizes, the lipid microenvironment of the membrane changes dramatically, and this directly affects embedded and peripheral proteins. The redistribution of PS alters local lipid packing, which drives clustering of transmembrane proteins and receptors (such as integrins, adhesion molecules, and glycoproteins) [50]. These clusters create microdomains where binding sites for circulating proteins are concentrated [51]. These newly exposed negative charges on PS attract plasma proteins that contain calcium-dependent binding motifs (e.g., the Gla domains of clotting factors) [40, 52]. Functionally, this means that even though the intrinsic protein-to-lipid ratio in the membrane does not change, the effective protein density at the surface increases as both membrane proteins and newly bound plasma proteins accumulate in PS-enriched regions. In essence, therefore it can be assumed that the PS flip reorganizes membrane proteins into prothrombotic clusters while simultaneously transforming the surface into a catalytic platform for the coagulation cascade.

Prothrombin, factor Xa, and factor Va assemble at PS-rich membrane domains, where negatively charged phospholipids provide the Ca²⁺-dependent binding sites required for formation and stabilization of the prothrombinase complex, thereby markedly enhancing thrombin generation [28, 53, 54]. Annexins and complement components are also recruited, which crosslink PS with membrane proteins and further consolidate a surface that is highly adhesive for clotting factors [7]. Factor Xa and Factor Va assemble on PS-rich microdomains to form the prothrombinase complex, which catalyzes the rapid conversion of prothrombin to thrombin [28]. Thrombin then cleaves fibrinopeptides from circulating fibrinogen to form fibrin monomers. Because the prothrombinase complex is assembled on PS-rich membrane surfaces (e.g. activated platelets or PS-exposed cells), subsequent fibrin formation (polymerization) begins in close proximity to those membrane surfaces [53]. The PS flip area is essential for the formation of the ternary prothrombinase-prothrombin complex (discovered by Shim et al. [55], and this anionic phospholipid layer provides a binding surface for the ternary complex assembly and is mainly provided by the membranes of activated platelets and endothelial cells [53].

Fibrin monomers polymerize into protofibrils and fibers after thrombin cleavage of fibrinogen; fibrinogen also binds adhesion receptors such as integrin αIIbβ3 when they are activated, which can localize and concentrate fibrin(ogen) at membrane surfaces [56, 57]. Although direct anchoring to PS-rich membrane fragments is less well quantified, these combined interactions bias fibrin deposition toward damaged (procoagulant) cell surfaces. This local enrichment of fibrinogen ensures that, once thrombin is generated, fibrin rapidly deposits at these procoagulant layers.

Another important molecule is fibronectin. It is a multifunctional adhesive glycoprotein that regulates clot formation, and orchestrates tissue repair and immune clearance [58]. Fibronectin also enhances thrombogenesis by cross-linking to fibrin [59] and promotes thrombus growth [60]. Fibronectin mostly binds to integrins, with α and β subunits located on the cell surface [61].

Although not a focus of this paper, Table 1 shows the various fibrinogen receptors on cells in the vasculature that interact with fibrinogen. In fact, cells of the vasculature are abundant in receptors for various inflammatory molecules. These molecules may interact with both healthy cells and the cellular debris resulting from cell death and inflammation, with both the functioning cells and debris creating binding surfaces for circulating inflammatory cells. One of the inflammatory molecules (serum amyloid A (SAA)) is known to be highly upregulated in various inflammatory diseases, and that is also well-recognized as an amyloid and amyloidogenic molecule [62, 63]. SAA proteins, particularly SAA1 and SAA2, mediate functions such as chemoattraction, cytokine induction, and modulation of lipid metabolism through receptors like Formyl Peptide Receptor 2 (FPR2), Toll-like receptor 4 (TLR4), and the receptor for advanced glycation end products (RAGE) [63, 64]. Binding of SAA to platelets is mediated through the integrin receptor αIIbβ3, whose ligands encompass a range of ECM proteins amongst which is fibronectin [64–66]. In particular, αIIbβ3 binds to talin, a cytoplasmic protein that is essential both for inside-out integrin activation and for linking integrin to the actin cytoskeleton [67]. Specifically, talin binds to the cytoplasmic tail of the integrin’s β3 subunit, with one binding site being a membrane-proximal region and another being the membrane-distal NPXY motif. The interaction of the amyloidogenic talin with αIIbβ3 is essential for proper activation of the integrin in platelets, thus playing a direct role in thrombosis and other amyloid-related processes [68, 69].

**Table 1:** Fibrinogen receptors and their associated cell types.

Cell type	Main fibrinogen-binding receptors	Comments
Platelets	Integrin αIIbβ3 (GPIIb/IIIa) [236] FPR2 [64]	The dominant fibrinogen receptor αIIbβ3 essential for platelet aggregation and thrombus formation. SAA also binds to this αIIbβ3 receptor [64]. Platelets also have FPR2 receptors. αIIbβ3 is preferentially and highly expressed on resting platelets with 60,000–80,000 copies per cell [237].
Endothelial cells	Integrins αvβ3 and α5β1 [15, 238] ICAM1 [239] FPR2	Mediate fibrinogen binding, endothelial adhesion, angiogenesis, and leukocyte interactions Fibrinogen receptors contribute to clot stability at vessel walls α5β1 on endothelial cells, atheroprone flow plus oxidized lipoproteins increases the high-affinity conformation of α5β1, making ECs more adhesive and proinflammatory [238] α5β1 binds fibrin and fibronectin [240] FPR2 binds SAA [64]
Leukocytes (esp. neutrophils, monocytes, macrophages)	Integrin αMβ2 [241] (Mac-1, CD11b/CD18) FPR2	Binds fibrinogen, supports leukocyte adhesion, migration, and immunothrombosis. FRP2 important in linking inflammation to coagulation [64]
Erythrocytes	Less well-defined but perhaps GPIIbIIIa [242]	Red cells can bind fibrinogen under inflammatory/prothrombotic conditions, but possibly not via a dedicated high-affinity receptor like platelets Not finally confirmed but GPIIbIIIa can possibly serve as fibrinogen binding sites. The presence of the GPIIbIIIa inhibitors reduces the amount of adsorbed fibrinogen, leading to a decrease in the hydrodynamic stability of RBC aggregates.

SAA also binds to FPR2 on neutrophils [70, 71]. FPR2 is present on human platelets, where it mediates responses to SAA and other ligands, contributing to platelet activation, aggregation, and thrombo-inflammatory signaling [64]. FPR2 is also abundantly expressed on neutrophils, monocytes, macrophages, and some lymphocyte subsets, where it regulates chemotaxis, phagocytosis, inflammatory processes [72]. Interestingly, SAA also binds to membrane surfaces that have undergone the PS flip [73].

In healthy individuals, decaying cells undergo rapid clearance by phagocytes [74]. Interestingly, although the number of apoptotic cells produced and lost daily is known to be some 50–70 billion in a healthy adult human, apoptotic cells are rarely observed [75]. This absence of numerous decaying cells, is due to the existence of a cellular process called efferocytosis that efficiently clears apoptotic cells [74]. Apoptotic cells that expose PS are typically and rapidly recognized by phagocytic cells (macrophages, etc.), via ‘eat-me’ signals [76]. This clearance helps to remove PS-exposed cells before they can nucleate coagulation.

### Damaged cell membranes can interact with various circulating inflammatory molecules

When PS-exposed procoagulant membranes encounter circulating inflammatory molecules (such as SAA, CRP, VWF, complement, or histones), these proteins bind to the negative surface, amplifying coagulation factor assembly and stabilizing the prothrombotic phenotype [7]. This interaction represents a major link between inflammation and pathological clotting [2]. Inflammatory mediators such as SAA, VWF, histones, complement proteins, and C-reactive protein can directly interact with PS-rich membranes, reinforcing their prothrombotic activity [34, 77, 78]. Negatively charged PS patches act as high-affinity binding sites for these proteins. SAA is for example an acute-phase protein that is inherently amyloid in nature. It can bind to membrane receptors, including αIIbβ3 and is capable of forming oligomers (e.g. in amyloidosis [79]), It is also strongly amyloidogenic under pathological conditions [80, 81]. As inflammatory molecules accumulate on PS-exposed membranes, they disrupt lipid microdomains, drive clustering of membrane proteins, and recruit coagulation factors such as prothrombin and factor Xa; together, these changes amplify and stabilize the procoagulant phenotype [82, 83].

In healthy physiology, PS exposure alone is insufficient to sustain thrombin generation because apoptotic cells are rapidly cleared. In inflammatory states, however, mediators such as CRP, histones, and serum amyloid A bind to PS-rich surfaces, protecting them from clearance and converting them into scaffolds for fibrin deposition. Thus, inflammatory molecules function as biochemical amplifiers, transforming normally transient PS-exposed membranes into persistent catalytic platforms (or prothrombotic seeding areas) that drive thromboinflammation. The resulting amyloidogenic and prothrombotic cellular seeding areas, we call fibrinaloid microclot complexes (FMCs). We suggest that various FMC phenotypes exist, and their characteristics are discussed later in this paper.

### Cellular senescence

Senescent cells accumulate in ageing phenotypes, but are also implicated in both noncommunicable and communicable diseases. For instance, cardiovascular disease, T2DM, and autoimmune conditions exhibit higher levels of cellular senescence [84–87]. Some viruses, such as SARS-CoV-2, and other pathogens are capable of inducing cellular senescence in a range of cell types [88, 89]. SARS-CoV-2 is known to induce endothelial [90] and leukocyte senescence [91, 92]. The senescent phenotype is characterized by an increase and dysregulation in pro-inflammatory molecules such as cytokines and chemokines, oxidative stress, dysregulation of growth factors, and procoagulant factors, such as VWF and TF [90, 93], specifically in hematological and vascular cells. Additionally, senescent cells and processes are also linked to amyloidogenesis [94, 95], and there are also indications that amyloid proteins and aggregates can induce cellular senescence [96, 97].

Hence, senescent processes can also induce thrombosis and implicate immune function. Senescent endothelial cells exhibit impaired nitric oxide production, increased reactive oxygen species, and loss of glycocalyx integrity, all of which predispose to endothelial barrier disruption and platelet adhesion [98, 99]. Furthermore, senescent cells often upregulate TF [100], thereby promoting a procoagulant membrane phenotype similar to that seen in apoptosis or eryptosis. The increase in procoagulant factors such as vWF and TF by senescent endothelial and other cells can exacerbate fibrin formation at prothrombotic surfaces in blood and at the vascular wall.

Importantly, the plasma membranes of senescent cells undergo biophysical remodeling, including altered lipid composition, membrane stiffening, and redistribution of proteins, contributing to impaired endocytosis, antigen presentation, and coagulation regulation [101–103]. These changes are compounded by loss of membrane lipid asymmetry and surface exposure of PS and annexins in some cases, as well as damage-associated molecular patterns (DAMPs) [100, 104], creating an environment primed for immune recognition, macrophage recruitment, and coagulation cascade activation. The persistence of senescent cells is particularly exacerbated in conditions where immune dysfunction is prominent, resulting in a decreased ability to recognize and remove senescent cells. Thus, cellular senescence represents a third major pathway – alongside apoptosis and necrosis – through which membrane alterations promote prothrombotic transformation of vascular and blood cell surfaces.

### Decaying cells can break down into smaller and smaller fragments and micropartices/microvesicles that become prothrombotic seeding areas

**Fig. 2:**
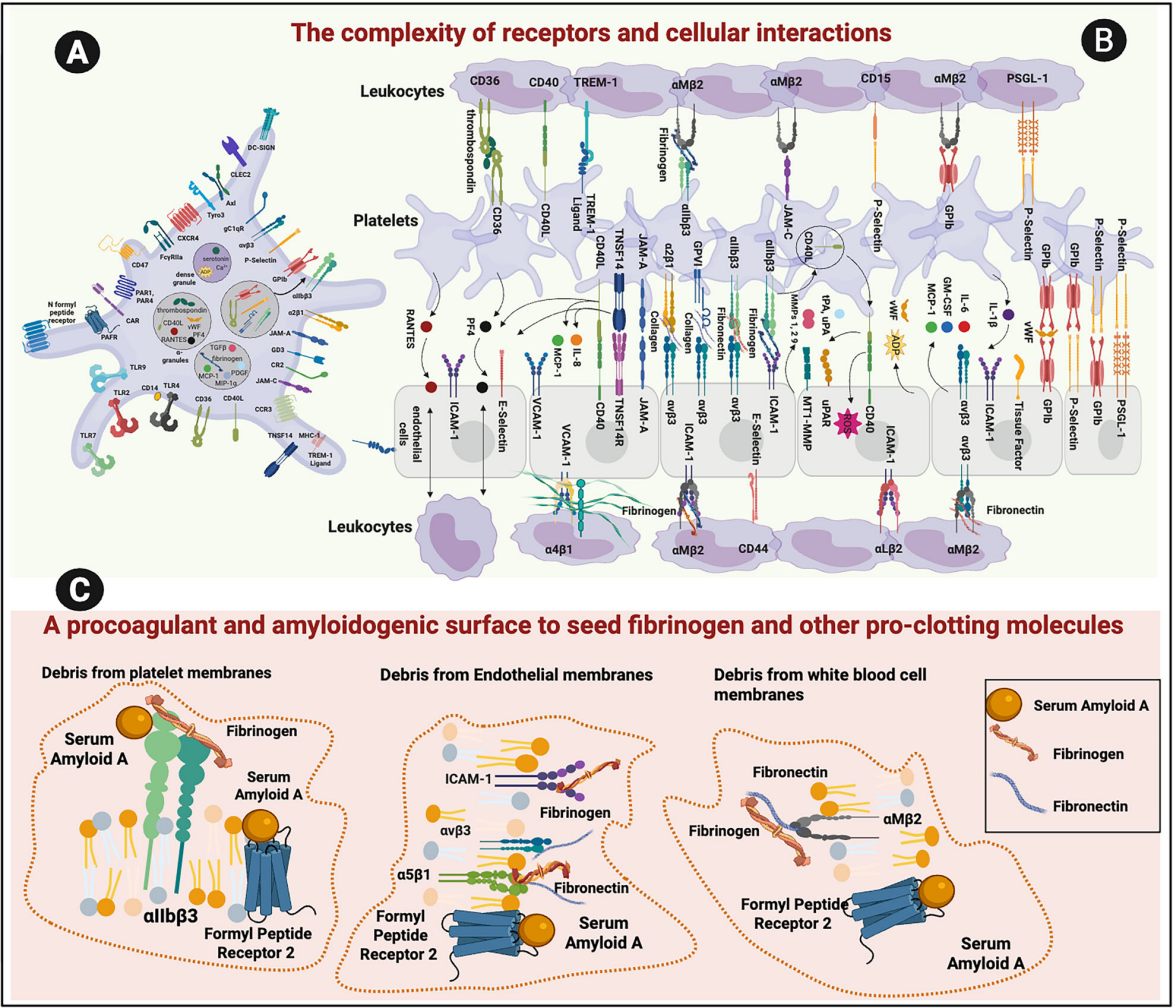
Integrated cellular and molecular mechanisms showing the complexity of receptors and cellular interactions between platelets, leucocytes and the vasculature. **A** Resting (non-activated) platelet with numerous low-affinity receptors on its surface. **B** The complexity of platelet membrane receptors during platelet activation where platelets can form complexes with other activated platelets, white blood cells and damaged endothelial cells. **C** The formation of a procoagulant and amyloidogenic surface to seed and fibrinogen and other pro-clotting molecules. Progressive membrane injury in platelets, leukocytes, and endothelial cells exposes phosphatidylserine and denatured or misfolded proteins, generating surfaces capable of binding fibrinogen, fibronectin, and serum amyloid A (SAA). These damaged membranes, enriched in amyloidogenic and negatively charged domains, act as catalytic scaffolds that seed procoagular surfaces. Created in https://BioRender.com.

As cells decay, their fragments or the resulting microparticles [105, 106] still carry these prothrombotic signals [107, 108] and can accumulate fibrinogen/fibrin and inflammatory molecules. Such circulating “micro” clots might not immediately be in high enough quantities or concentrations to initiate a true clot. However, these smaller entities may be able to associated with fibrin networks. It is e.g. known that microparticles (that are also prothrombotic entities) can accumulate and build into fibrin networks [109–111]. Zubairova et al. in 2015 showed that microparticles accelerate fibrin polymerization and support formation of more dense fibrin clots that resist fibrinolysis. Such microparticles drive faster thrombin generation, impact thrombin-mediated kinetic effects of fibrin formation, and impacts fibrin structure and properties. Fibrin clots formed in the presence of microparticles, contain 0.1–0.5-μm size granular and CD61-positive material on fibers, suggesting that platelet-derived microparticles attach to fibrin [109] (see Fig. 2).

### Microclot complexes are not ‘just’ microparticles

Although microparticles can bind to fibrin(ogen) [109, 110, 112], and there is evidence that FMCs can contain microparticles, we would stress that they are not synonymous for a variety of reasons:

1. Size. Microparticles are typically in the range 0.05–1 μm [113–120], and exosomes are in the lower range of these [121], whereas FMCs are typically in the range 1–200 μm [122, 123].
2. Number. Microparticles of the above size range can be present in plasma in very large numbers, values quoted ranging from 8.10^6^ to 4.10^9^/mL [113]; ~3.10^6^/mL has been stated just for platelet-derived microparticles [124]. Orozco and coworkers found ~10^8^ microparticles/mL [125], Albert and co-workers over 10^6^/mL [126] and Chandler and coworkers, numbers from 3.10^6^ to 10^8^/mL [113]. By contrast, FMC greater than 1 μm in equivalent diameter are commonly present in numbers with a median below 1000/mL [122] and a maximum value around 6.10^5^/mL, even in pathological conditions.
3. Composition. The composition of microparticles simply reflects the composition of the cells from which they originate, and these cellular origins typically include platelets [110, 127, 128], erythrocytes [129], leukocytes [130, 131], and endothelial cells [131–134]. Unsurprisingly, their origin affects their thrombotic potential [110, 135–137] as well as reflecting the diseases with which they are associated [138, 139]. We note too the possibility that nanoplastics may also contribute to a pathological microparticle burden [140, 141] as they themselves are amyloidogenic [142]. The same issues pertain for similarly sized particulate matter ingested via air pollution (e.g. refs. [143, 144]). Importantly, because these items are essentially insoluble they too can contribute to the blockage of the microcirculation that underpins so much of the pathology of FMCs. However, microparticles themselves are not specifically enriched in fibrin(ogen) albeit that they can bind it. We note that by contrast the FMCs are dominated by fibrin(ogen) subunits [145–147] and are significantly enriched in amyloidogenic proteins [148, 149]. In addition, environmental and endogenous metals may contribute to pathological microparticle burdens and to thrombo-inflammatory processes [150, 151]. Transition metals such as iron, copper, and other redox-active metals can catalyze oxidative reactions (e.g. Fenton chemistry) that promote protein misfolding, lipid peroxidation, and structural modification of plasma proteins including fibrinogen [152]. Such oxidative processes may facilitate amyloidogenic aggregation and stabilize FMCs particularly in inflammatory environments where metal dysregulation and oxidative stress are common [153]. In addition to soluble metal-driven oxidative processes, particulate crystalline structures may also contribute to pathological microparticle burdens that promote thrombo-inflammation. Micro-crystalline debris, calcium-derived crystalline deposits, and uric acid crystals that form during inflammatory or metabolic stress can present highly ordered surfaces capable of binding plasma proteins and coagulation factors [154, 155]. These crystalline interfaces may possibly also act as nucleation platforms for protein misfolding and amyloidogenic aggregation, promoting the formation and stabilization of FMCs within inflammatory vascular environments.
4. Causality. It would seem that lipid microvesicles will bind fibrin(ogen) in microparticles, but that actual clotting in the FMCstraps other things, including microparticles. Consequently, the order of adding fibrin in the two structures might be opposite.

### Biochemical characteristics of molecules that can associate with decaying membranes or act as prothrombotic seeding areas

Many pro-inflammatory molecules that have previously been found to accumulate on procoagulant cellular membrane debris have an inherent amyloidogenicity; for a review see ref. [148]. Any protein’s amyloidogenicity can be determined by using predictive bioinformatics tools such as AmyloGram [156]. Such molecules may also themselves interact with circulating fibrinogen and plasma proteins when added to healthy plasma. Examples of such interactions are SAA, complement, as well as interleukins [62, 157, 158]. Computational tools such as AmyloGram and related predictive algorithms enable the identification of amyloidogenic regions within protein sequences by detecting structural features that favor cross-β sheet formation. These approaches are particularly useful for identifying aggregation-prone segments in proteins that participate in inflammatory and coagulation pathways. In the context of FMCs, several circulating proteins have been reported to contain such amyloidogenic regions, including fibrinogen, serum amyloid A, complement components, and certain viral proteins. Many of these proteins are upregulated during inflammation and may interact with fibrinogen or damaged membrane surfaces, facilitating cross-seeding and the formation of β-sheet–rich aggregates. Predictive bioinformatic tools therefore provide an important framework for identifying candidate proteins that may contribute to FMC formation and stability in thrombo-inflammatory conditions [148, 149, 159–161].

As mentioned in previous paragraphs, SAA is an abundant inflammatory molecule and has numerous receptors on cell membranes [63, 64, 66, 70, 80, 162]. SAA and fibrinogen share receptors, suggesting that co-binding, or adjacent receptor binding to the receptors left on the debris occurs. This provides evidence that these seeding areas are not only prothrombotic but also amyloidogenic. We have found SAA to be significantly upregulated in various inflammatory diseases [163–166]. Proteomic analyses show that unusually amyloidogenic proteins are enriched in such clots, probably by actually being incorporated into cross-beta elements of the developing fibrils [148, 149].

In purified fibrin(ogen) systems, defined amyloid fibrils from SARS-CoV-2 spike peptides exert segment-specific effects on clot dynamics and lysis [167]. Westman and coworkers showed that fibrils from spike601 (aa601–620) delayed thrombin-driven fibrin formation by sequestering fibrinogen without impairing subsequent fibrinolysis, whereas fibrils from spike685 (aa685–701; just C-terminal to the furin site) promoted denser clot architecture and yielded plasmin-resistant residues in a dose-dependent manner [167]. Fluorescence co-localization and TEM indicated that spike685 fibrils co-assemble with fibrin into granular/fibrous aggregates that persist after tPA/plasmin treatment, consistent with impaired fibrinolysis [168].

In the case of fibrinogen, the fibrinogen α-chain subunit, and specifically its αC domain, is often considered to be the most amyloidogenic [169, 170], on the grounds that hereditary mutations in the fibrinogen Aα (FGA) gene can lead to Fibrinogen Alpha Chain (AFib) amyloidosis [171, 172].

These findings provide a concrete biochemical mechanism by which spike-derived amyloid, particularly spike685, could seed fibrinolysis-resistant (micro) clots relevant to specifically long COVID pathophysiology. While the data derive from controlled in vitro assays mostly do not model whole-blood shear [173] or cellular clearance, they identify an aggregation-prone spike segment with clear potential to bias clot structure toward persistent, prothrombotic states [167].

### A healthy circulation avoids uncontrolled SAA– or fibrinogen–receptor binding

There are various mechanisms whereby receptor binding does not randomly activate in the presence of possible ligands like SAA and fibrinogen. In healthy circulation, random or uncontrolled binding of SAA or fibrinogen to their receptors is prevented by several protective mechanisms. Platelet integrins such as αIIbβ3 remain inactive until specifically triggered by inside-out signaling, ensuring that fibrinogen cannot bind under basal conditions [17]. The intact endothelial glycocalyx, together with continuous release of nitric oxide (NO) and prostacyclin (PGI₂), enforces an anti-adhesive and anti-thrombotic vascular surface [174]. Circulating acute-phase proteins such as SAA are normally sequestered in high-density lipoprotein (HDL) complexes, limiting their free availability for receptor engagement [175, 176]. Finally, maintenance of membrane asymmetry by flippases keeps PS restricted to the inner leaflet, thereby preventing exposure of the negatively charged surfaces that otherwise act as procoagulant platforms [17]. See Tables 2, 3 and 4 for summaries.

**Table 2:** Regulatory processes to prevent receptor activation.

Regulatory layer	Mechanism	Effect on SAA/fibrinogen/receptors	Key references
Receptor conformation	Platelet integrin αIIbβ3 is kept in a low-affinity state until “inside-out” signaling (e.g., via thrombin, ADP) activates it	Prevents fibrinogen and SAA from binding under resting conditions	[243]
Endothelial anti-adhesive surface	Endothelial glycocalyx (heparan sulfates, proteoglycans) blocks receptor access; NO and PGI₂ secretion suppress platelet activation	Prevents SAA and fibrinogen interaction with receptors on healthy endothelium and platelets	[174, 244]
Plasma binding partners	SAA is mostly HDL-bound in health; fibrinogen requires thrombin cleavage to reveal αIIbβ3 binding sites	Circulating ligands are “shielded” from receptor engagement	[245]
Membrane lipid asymmetry	Phosphatidylserine (PS) is restricted to inner leaflet by flippases	Prevents formation of procoagulant binding sites for clotting factors and SAA	[17]

**Table 3:** Protective mechanisms preventing receptor–ligand interactions in healthy circulation and their alteration during inflammation.

Protective mechanism (healthy circulation)	Change during inflammation/disease
Integrins (e.g., αIIbβ3) inactive → low-affinity state maintained until platelet inside-out signaling activates them [243, 246]	Platelet activation by thrombin, ADP, thromboxane A₂, or cytokines triggers conformational change of αIIbβ3 → high-affinity fibrinogen and SAA binding [246]
Endothelial glycocalyx + NO/PGI₂ enforce anti-adhesion, maintain vascular quiescence [174]	Glycocalyx degraded by ROS, proteases, and inflammatory enzymes; reduced NO/PGI₂ signaling → adhesion molecules and receptors exposed
SAA sequestered in HDL complexes under baseline conditions, minimal free circulating SAA [247]	Acute-phase response: SAA upregulated 1000-fold and dissociates from HDL [248] Large pool of free can then potentially SAA binds receptors and fibrinogen.
PS restricted to inner leaflet by flippases maintains lipid asymmetry, no catalytic surface for coagulation [17, 22]	In apoptosis/activation: scramblase activation + flippase inhibition; PS externalization, generating negatively charged catalytic surfaces for factor binding [17]

**Table 4:** Safety mechanisms of SAA in health and disease.

Protective partner (health)	Mechanism of “safety”	Status in health (SAA levels < 5 mg/L)	What changes in inflammation (SAA up to 1000 mg/L)	References
HDL (apoA-I containing particles)	Major carrier, prevents SAA functioning as an inflammatory molecule	SAA tightly bound to HDL; free SAA negligible	HDL composition altered; apoA displaced by SAA (“acute-phase HDL”) with reduced protective function; free SAA increases	[249, 250]
Apolipoproteins	ApoA stabilize HDL, reducing SAA exposure	ApoA is abundant; maintain HDL’s anti-amyloid function	ApoA levels fall; displaced by SAA, exposure of hydrophobic domains; ApoE overwhelmed.	[245]
Lipid components (phospholipids, cholesterol esters)	SAA intercalates into HDL phospholipid monolayer, hydrophobic residues shielded	Normal lipid ratios keep SAA soluble within HDL	Acute-phase HDL lipid ratios altered; SAA less shielded and more aggregation-prone	[249, 251]

### The heterogeneity of complex hydrophobic, amyloid-containing biological structures, including lipofuscin, atherosclerotic plaques, and fibrinaloid microclot complexes and macroclots

Most simple biological structures are more or less homogeneous, as evolution has selected them to maintain specific functions and hence structures, although we recognize the ability of various ‘metamorphic’ [149] or ‘fold-switching’ [177–180] proteins to adopt multiple stable conformations In particular, prions and other proteins can adopt stable, amyloid states that often self-organize into insoluble fibrils of fairly similar diameters in the range 10–20 nm governed by the length of the cross-beta motif that characterises such amyloid.

Many classical amyloidoses have been described [181, 182]. For the present context, however, we emphasize our finding [168] that fibrinogen can interact with amyloidogenic inflammatory molecules either directly in circulation or at sites where fibrinogen receptors are expressed. These interactions give rise to insoluble, misfolded, amyloidogenic protein complexes that can be observed in both whole blood and platelet-poor plasma and they stain positively with the fluorogenic dyes like ThT. We have termed these misfolded and amyloidogenic heterogenous deposits, “microclots” or FMCs. Moreover, the clotting cascade, culminating in the conversion of prothrombin to thrombin, can be triggered, resulting in true fibrin complexes that have also adopted an anomalous, misfolded and potentially amyloidogenic structure.

Cross-seeding of amyloidogenic proteins leads to fibrils containing a very large variety of different proteins [148]. The hydrophobic patches of amyloids also bind strongly to lipids, something important to their cytotoxicity [183–185]. The presence of metals such as iron that can catalyze the Fenton reaction [150, 186] is also likely since many of the proteins are highly oxidized (see also for atherosclerotic plaques [187–189]).

Another set of structures that also contain lipids, amyloid proteins and DNA in a very heterogeneous layout is represented by lipofuscin [190–193], a complex pigment found in the lysosomes of ageing cells [194–196].

Yet another complex set of structures involving lipids and amyloidogenic proteins (as well as other materials, not least unliganded iron ions that catalyse oxidations [150]), is represented by atherosclerotic plaques [194–197], that are obviously highly important in the generation of myocardial infarctions and other vascular problems. Amyloid(ogenic) proteins that they are known to contain include alpha_1_-antitrypsin [198, 199], serum amyloid A [200–202], apolipoprotein A1 [203–205] and amyloid-beta [206, 207]. Strikingly, the first three of these have also been found in heterogenous deposits or FMCs [62, 145, 147], consistent with their appearance in and contributions to a variety of thrombotic diseases [160] and the general biophysics underlying cross-seeding [148, 149]. The same is true for the kinds of heterogeneous plaque found in Alzheimer’s dementia [208].

Finally, we recently established that the macroclots isolated from individuals following an ischemic stroke are also amyloid in nature [209], with the heterogeneity being especially evident from the comparison of brightfield and fluorescence images [210]. Consequently, characterization of this kind of heterogeneity becomes important if we are to understand its significance.

### Characterizing (heterogenous) prothrombotic complexes

Characterization of these (heterogenous) proinflammatory circulating complexes in the form of “micro”clots remains challenging. We have been using Thioflavin T (ThT), which is a benzothiazole dye that remains non-fluorescent when free in solution because its two aromatic rings can rotate freely around a central single bond. Fluorescence occurs when ThT binds to a surface that restricts its rotational freedom, locking the dye into a planar conformation. This mechanism underlies its widespread use as an amyloid probe: aligned within the repetitive grooves of cross-β sheet fibrils, ThT becomes immobilized and emits a strong fluorescent signal [211–213] However, the same mechanism also means that ThT can interact with other repetitive or rigid hydrophobic surfaces, potentially including lipid assemblies [214], nucleic acids [215] and damaged or misfolded proteins [216–218].

In complex biological environments, ThT fluorescence can therefore also indicate the presence of structured hydrophobic binding sites. We have previously shown that we can induce ThT binding to purified fibrinogen after addition of SAA [62, 163], LPS, LTA, gingipains [219–221] and also spike protein [222, 223]. SAA is, of course, a known amyloidogenic molecule [162, 224, 225] and is upregulated in the circulation in various inflammatory diseases [163, 164, 226]. The SARS-CoV-2 spike protein also has amyloidogenic potential and its spike protein (especially in the presence of LPS or amylin) [227] may form aggregates, and Amytracker probes show small aggregates [159, 228, 229].

In summary, ThT fluorescence thus arises when its rotational freedom is restricted, most strongly when the dye intercalates into the repetitive grooves of cross-β sheet amyloid fibrils. While ThT is known to also bind certain non-amyloid surfaces, in complex biological systems such as plasma or damaged cell membranes, its signal is most informative when it highlights regions where amyloidogenic proteins may accumulate. Proteins like fibrinogen, SAA, or other aggregation-prone inflammatory molecules can adopt β-sheet–rich conformations upon interaction with perturbed membranes, creating surfaces that both restrict ThT rotation and report amyloidogenic potential. Thus, even though ThT can bind to hydrophobic structures in general, in the prothrombotic context a ThT signal may suggest the presence of interactions between proteins with amyloidogenic potential and damaged membrane surfaces or such amyloidogenic inflammatory molecules interacting with fibrin(ogen). Both these scenarios provide evidence for the presence of complexes with amyloid-like properties in inflammatory and post-viral diseases.

### Challenges in detecting FMCs for diagnostic purposes

Despite increasing evidence for the presence of FMCs in inflammatory conditions, several challenges remain for their routine diagnostic detection. FMCs are structurally heterogeneous assemblies composed of fibrin(ogen), inflammatory proteins, and NETs cellular debris, and lipid components, resulting in substantial variability in size, composition, and abundance between individuals and disease states. This heterogeneity complicates the development of standardized assays and thresholds for clinical interpretation. In addition, commonly used amyloid probes such as ThT detect β-sheet–rich structures but are not entirely specific for amyloid fibrils, as they may also bind other ordered hydrophobic surfaces present in complex biological samples. Furthermore, because FMCs represent dynamic assemblies that may evolve during inflammation or coagulation, single-time-point measurements may not fully capture their biological significance. An additional challenge, but also an important opportunity, lies in identifying and quantifying the specific FMC phenotypes that exert the greatest pathological impact, as distinguishing the most biologically active or disease-driving complexes could substantially enhance the diagnostic and prognostic value of FMC measurements; importantly, pathogenicity may depend not only on FMC abundance or size, but also on the molecular composition of entrapped inflammatory mediators and NET-derived components. Addressing these analytical and methodological challenges will be essential for translating FMC detection into robust diagnostic platforms.

### Summarizing our thoughts on the various phenotypes of circulating prothrombotic complexes (we termed fibrinaloid microclot complexes (FMCs)) that may drive thrombo-inflammation on β-sheet rich and amyloidogenic surfaces

**Fig. 3:**
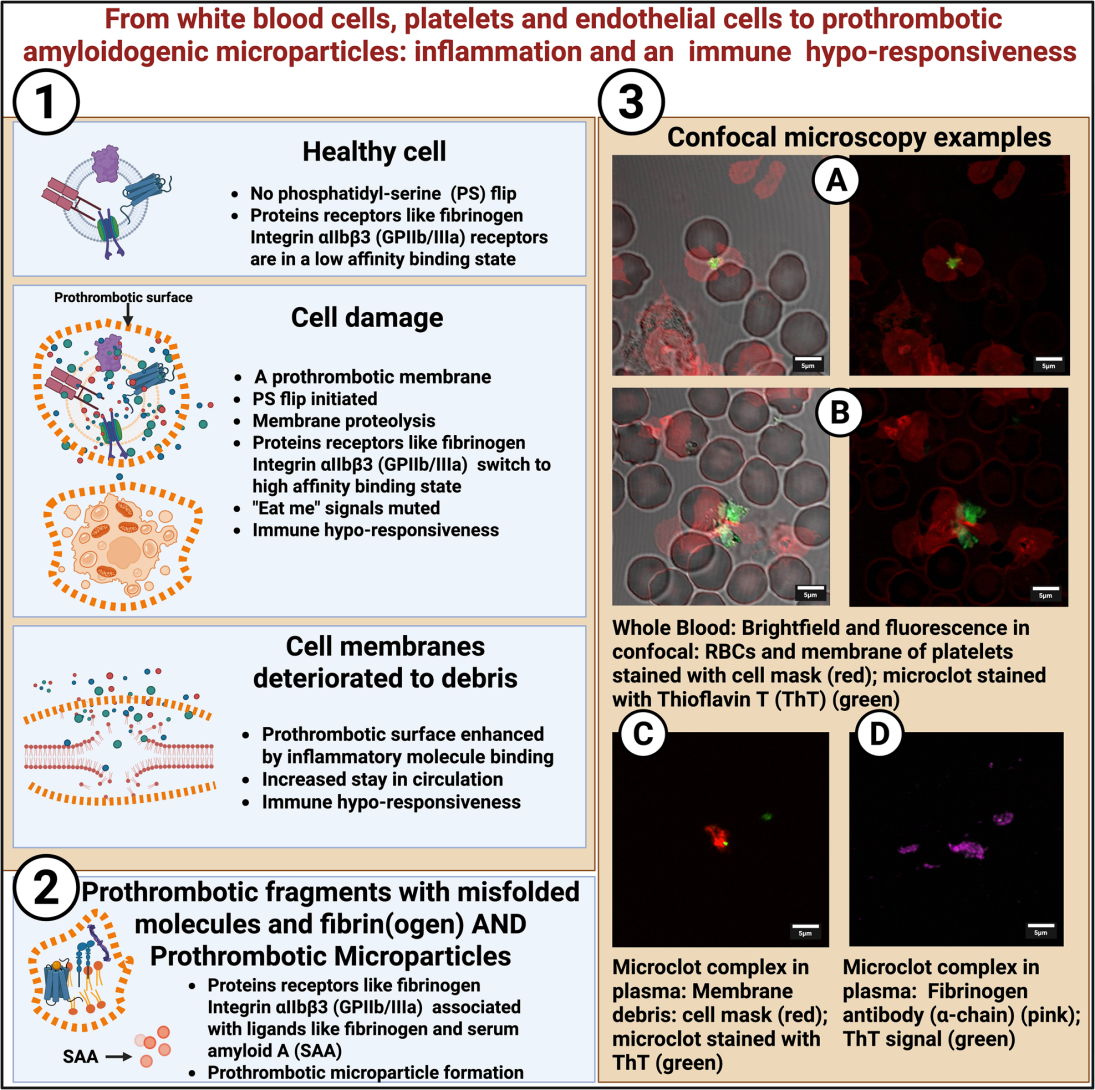
The roadmap to circulating prothrombotic complexes (FMCs) that may drive thrombo-inflammation on β-sheet rich and amyloidogenic surfaces via the cellular debris pathway. (**1**) *Healthy and damaged cells:* In healthy membranes, phosphatidylserine (PS) is internalized and integrin αIIbβ3 (GPIIb/IIIa) remains in a low-affinity state. Upon injury, PS externalizes (“PS flip”), receptors activate, and membranes become prothrombotic and immune-silent. Progressive damage generates debris that binds inflammatory molecules. (**2**) *Prothrombotic fragments and microparticles:* Exposed receptors interact with serum amyloid A (SAA) and fibrin(ogen), forming β-sheet–rich, amyloidogenic scaffolds that seed fibrinaloid microclots (FMCs). (**3**) *Confocal microscopy:* **A**, **B** Whole blood: red blood cells and platelets stained with cell mask (red); amyloid signal detected with Thioflavin T ((ThT) green)). **C**, **D** Platelet-poor plasma (PPP): membrane debris or fibrin(ogen) α-chain antibody (pink) co-localizing with ThT-positive FMCs. Created in https://BioRender.com.

**Fig. 4:**
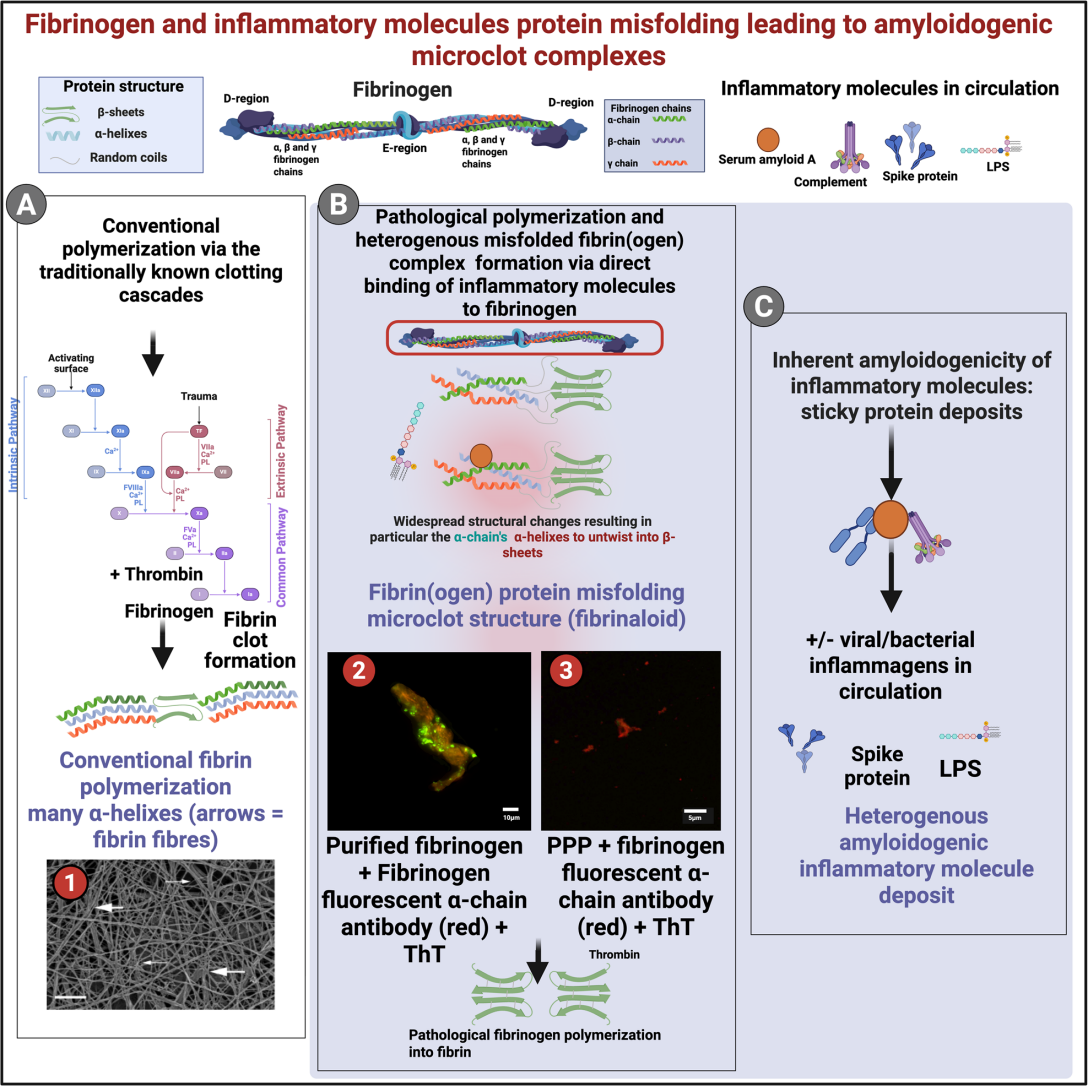
The pathway to circulating prothrombotic complexes (FMCs) that may drive thrombo-inflammation on β-sheet rich and amyloidogenic surfaces via the fibrinogen and inflammatory molecules pathway. **A** *Conventional fibrin formation:* Thrombin converts fibrinogen into fibrin monomers that polymerize into α-helical fibrin fibers, forming organized, degradable clots. **B** *Pathological polymerization:* Inflammatory molecules bind fibrinogen, inducing structural misfolding and β-sheet formation. These aberrant fibrin(ogen) complexes (“fibrinaloid” structures) are visualized with Thioflavin T ((ThT) green)) and fluorescent fibrinogen α-chain antibody (red) in purified fibrinogen and platelet-poor plasma (PPP). **C** *Amyloidogenic inflammatory molecules:* Circulating inflammagens such as spike protein, lipopolysaccharide (LPS), and serum amyloid A (SAA) are intrinsically amyloidogenic and can bind fibrinogen to form heterogeneous prothrombotic deposits. Created in https://BioRender.com.

**Fig. 5:**
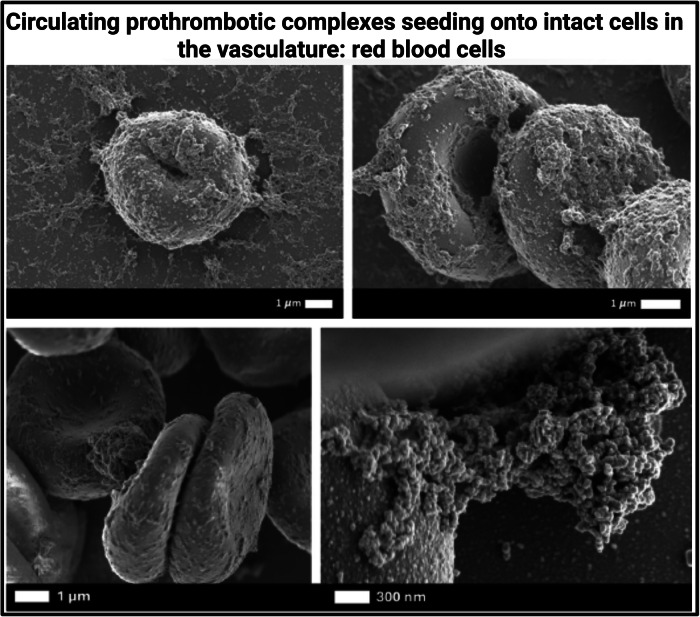
The pathway to circulating prothrombotic complexes (FMCs) (and/or microparticles) that may drive thrombo-inflammation on β-sheet rich and amyloidogenic surfaces via the seeding onto functional cells of the vasculature pathway (example given: seeding onto red blood cells: scanning electron micrographs of whole blood).

**Fig. 6:**
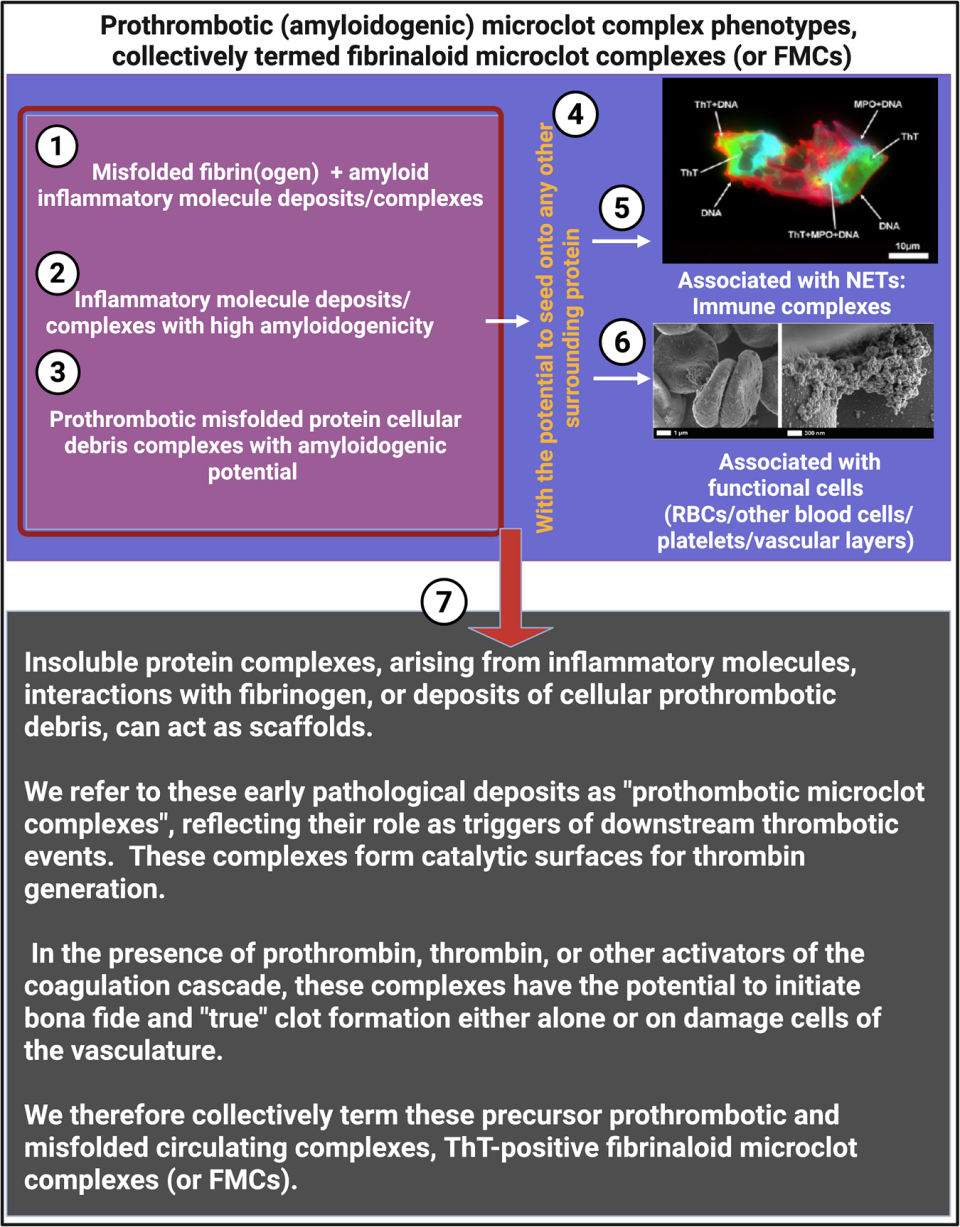
The pathway to circulating prothrombotic complexes (FMCs) that may drive thrombo-inflammation on β-sheet rich and amyloidogenic surfaces. From microclot complexes to anomalous fibrin dense matted deposits. (**1–3**) Progressive stages of pathological complex formation, beginning with misfolded fibrin(ogen) bound to amyloidogenic inflammatory molecules, evolving to mixed inflammatory deposits and misfolded cellular debris with prothrombotic potential. (**4–6**) FMCs are associated with neutrophil extracellular traps (NETs) and immune complexes (ThT⁺/MPO⁺/DNA⁺) and can interact with functional blood cells, including red blood cells and platelets, as shown by fluorescence and scanning electron microscopy. (See micrograph at (5) taken from [230]. (**7**) These insoluble protein complexes, arising from inflammatory molecules or fibrinogen interactions, act as scaffolds for thrombin generation and may seed further amyloid or fibrin deposition. In the presence of prothrombin or thrombin, they can initiate bona fide clot formation on vascular or cellular surfaces. Collectively, these Thioflavin T (ThT)-positive misfolded circulating complexes are termed fibrinaloid microclot complexes (FMCs). Created in https://BioRender.com.

We suggest that circulating prothrombotic complexes (or fibrinaloid microclot complexes (FMCs)), drive thrombo-inflammation can be classified into several distinct phenotypic forms (see Figs. 3–6).

Our suggested phenotypes are:

#### Cell-derived debris

These are characterized by dying cells or large fragments of damaged or apoptotic endothelial cells, erythrocytes, platelets, and leukocytes. These cellular debris retain intact membrane patches with exposed phosphatidylserine and clustered membrane proteins, serving as initial scaffolds for clotting factor binding. Furthermore, proteins in decaying membranes become β-sheet rich, as they unfold.

#### Subcellular vesicles and microparticles

Apoptotic bodies and microparticles (0.1–1 μm), derived from shedding or fragmentation of the parent cell membranes are rich in membrane proteins, PS patches, and receptors; capable of binding fibrinogen, prothrombinase, and annexins.

#### Nucleoprotein immune complexes

Neutrophil extracellular traps (NETs) are DNA–protein assemblies composed primarily of extracellular chromatin, histones, and neutrophil enzymes such as myeloperoxidase (MPO). These structures can act as nucleation sites for fibrin(ogen), recruiting additional inflammatory and coagulation factors to form prothrombotic complexes. As shown in Fig. 6, NETs can associate with pre-existing amyloidogenic seeding surfaces or independently promote FMC formation. NETs are frequently observed in inflammatory conditions including COVID-19, Long COVID [230, 231], and cancer, where they contribute to persistent vascular inflammation and immunothrombosis. Recent findings demonstrate that FMCs in Long COVID frequently co-localize with NETs, visualized using THT, myeloperoxidase (MPO), and Hoechst staining in PPP. This association likely stabilizes FMCs, impairs fibrinolysis, and amplifies endothelial injury, positioning NET-associated FMCs as key drivers of immunothrombosis and potential diagnostic targets [1, 230, 232].

#### Plasma protein aggregates associated with amyloidogenic inflammatory molecules

Fibrin(ogen) that associate with SAA, VWF and other inflammatory circulating proteins, such as spike protein, can co-aggregate or associate with fibrin(ogen) and thus can result in amyloid-like conformations. The rational for this statement is because types of proteins or peptides are either prone to cross-β sheet formation, or a amyloid molecule themselves (e.g. SAA).

Together, these phenotypic forms represent a pathological mode of alteration that is distinct from canonical thrombin-driven clotting, which normally yields fibrin clots that are susceptible to fibrinolysis and generate breakdown products such as D-dimer. These immune-thrombotic complexes are rather circulating signaling entities that show significant ThT-binding capacity and that are resistant to classical fibrinolytic processes due to their composition. We therefore suggest that these structures act as prothrombotic seeding areas that recruit fibrinogen, fibrin, and inflammatory molecules, forming the initial scaffolds of “micro” clots. At this stage, they may not yet contain sufficient material to develop into a fully formed thrombus, but they can be detected using markers for fibrin(ogen) or associated inflammatory molecules. The kinetics of their formation, and how their composition evolves over time, remain largely unexplored. Among circulating amyloidogenic proteins, SAA is one of the most prominent and shares several receptors with fibrinogen, suggesting a mechanistic link between inflammation, amyloid formation, and early thrombotic signaling.

### Comorbidities that may predispose to FMC formation

Several comorbid conditions may increase the risk of forming FMCs because they promote chronic inflammation, oxidative stress, endothelial injury, or dysregulation of coagulation pathways. Metabolic disorders such as T2DM and obesity are associated with persistent inflammatory signaling, glycation of plasma proteins, and endothelial dysfunction, all of which can enhance fibrinogen modification and protein aggregation. Cardiovascular diseases and atherosclerosis are similarly characterized by oxidative stress, iron dysregulation, and inflammatory activation, creating environments conducive to amyloidogenic protein interactions and aberrant clot formation. Autoimmune and chronic inflammatory diseases, including conditions such as rheumatoid arthritis or systemic inflammatory syndromes, may further elevate circulating acute-phase proteins such as serum amyloid A, thereby increasing the likelihood of amyloidogenic fibrin(ogen) interactions. Finally, infectious and post-viral conditions, including SARS-CoV-2 infection and Long COVID, are associated with persistent thrombo-inflammation, endothelial damage, and immune activation, all of which may favor the formation and persistence of FMCs. Notably, many of these conditions are also associated with increased neutrophil activation and NET formation, which may act as upstream drivers of immunothrombosis by providing extracellular DNA–histone scaffolds that bind fibrinogen, inflammatory mediators, and coagulation factors, thereby facilitating the formation and persistence of FMCs; consistent with this view, NET structures have been shown to be structurally associated with circulating FMCs in Long COVID and may participate in inflammatory feedback loops that sustain neutrophil activation [232]. Together, these comorbidities highlight how FMC formation may represent a downstream structural manifestation of systemic inflammatory and procoagulant states. Together, these comorbidities highlight how FMC formation may represent a downstream structural manifestation of systemic inflammatory and procoagulant states. Comorbidities are also evident in the disorders of the microcirculation that may be observed using techniques such as nailfold capillaroscopy, laser Doppler and laser speckle methods, and thermal imaging [233–235].

### Translational relevance of fibrinaloid microclot complexes

FMCs represent a structurally and mechanistically distinct class of circulating thrombo-inflammatory assemblies which has direct translational implications. Since FMCs are enriched in fibrin(ogen), SAA, and other amyloidogenic inflammatory proteins, they may serve as early biomarkers of thrombo-inflammatory disease states. Their detection in platelet-poor plasma using fluorescence probes such as ThT or related amyloid-binding dyes, provides a potential route for rapid laboratory diagnostics, enabling identification of pathological clotting signatures that may not be captured by conventional coagulation tests such as D-dimer or prothrombin time. In parallel, the molecular composition of FMCs suggests several therapeutic opportunities. Interventions that prevent protein misfolding, disrupt amyloid cross-seeding, restore fibrinolytic activity, or block interactions between inflammatory proteins and fibrinogen may reduce FMC formation or promote their clearance. Furthermore, targeting upstream drivers such as phosphatidylserine exposure, inflammatory mediators, or circulating amyloidogenic proteins could limit the generation of procoagulant surfaces that seed FMC formation. Emerging analytical approaches, including imaging flow cytometry coupled with fluorogenic amyloid probes, may allow quantitative determination of FMC abundance and phenotypic diversity in plasma, while therapeutic strategies targeting upstream stabilizing structures such as NETs may represent a promising strategy to reduce FMC formation, accumulation, and stability. Collectively, these observations position FMCs not only as mechanistic indicators of thrombo-inflammation but also as actionable translational targets for both diagnostics and therapeutic intervention in inflammatory and post-infectious vascular syndromes.

Thus in conclusion, we suggest that Insoluble protein complexes, arising from inflammatory molecules, interactions with fibrinogen, or deposits of cellular prothrombotic debris, can act as scaffolds. We refer to these early pathological deposits as circulating “prothombotic microclot complexes”, reflecting their role as triggers of downstream thrombotic events. These complexes form catalytic surfaces for thrombin generation. In the presence of prothrombin, thrombin, or other activators of the coagulation cascade, these complexes have the potential to initiate bona fide and “true” clot formation either alone or on damage cells of the vasculature. We therefore collectively term these precursor prothrombotic and misfolded circulating complexes, ThT-positive fibrinaloid microclot complexes (or FMCs).

## Data Availability

All micrograph data presented in figures are available. All requests for materials should be addressed to Etheresia Pretorius.
